# Influence of the Synthesis Conditions on the Morphology and Thermometric Properties of the Lifetime-Based Luminescent Thermometers in YPO_4_:Yb^3+^,Nd^3+^ Nanocrystals

**DOI:** 10.1021/acsomega.2c03990

**Published:** 2022-08-24

**Authors:** Kamila Maciejewska, Lukasz Marciniak

**Affiliations:** Institute of Low Temperature and Structure Research, Polish Academy of Sciences, Okólna 2, 50-422 Wroclaw, Poland

## Abstract

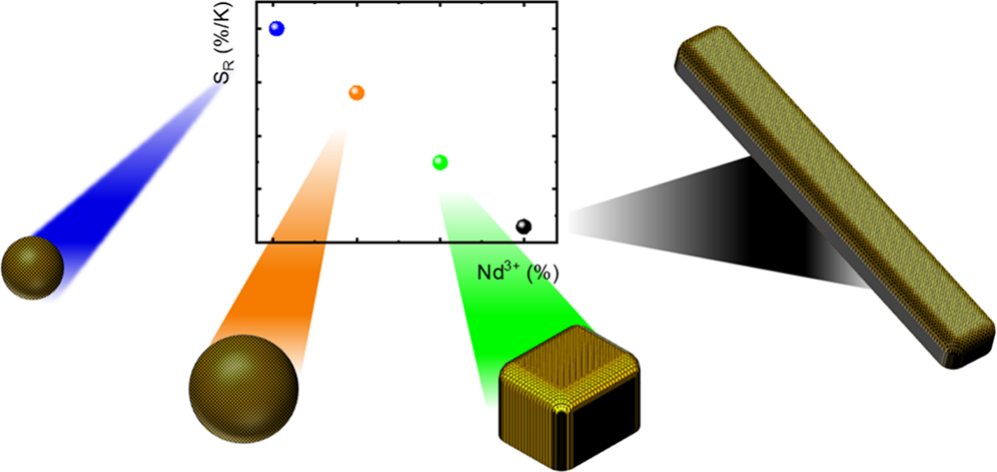

An increase in the accuracy of remote temperature readout using luminescent thermometry is determined, among other things, by the relative sensitivity of the thermometer. Therefore, to increase the sensitivity, intensive work is carried out to optimize the host material composition and select the luminescent ions accordingly. However, the role of nanocrystal morphology in thermometric performance is often neglected. This paper presents a systematic study determining the role of synthesis parameters of the solvothermal method on the morphology of YPO_4_:Yb^3+^,Nd^3+^ nanocrystals and their effect on the lifetime of Yb^3+^ ion-based luminescent thermometer performance. It was shown that by changing the RE^3+^:(PO_4_)^3–^ ratio and the concentration of Nd^3+^ ions, the size, shape, and aggregation level of the nanocrystals can be modified changing the thermometric parameters of the luminescent thermometer. The highest relative sensitivity was obtained for the low RE^3+^:(PO_4_)^3–^ ratio and 1% Nd^3+^ ion concentration.

## 1 Introduction

Materials that exhibit emission of electromagnetic radiation under nonthermal stimuli called phosphors are commonly used primarily in the lighting industry.^1−4^ However, in recent years, they have been increasingly used in many, much more demanding applications. One of the most interesting is the exploitation of luminescence for remote sensing of physical or chemical quantities of the medium in which they are located, including luminescence thermometers, manometers, pH meters, etc.^5−10^ Among these techniques, luminescence thermometry is undoubtedly the most strongly developed.^11−19^ It enables remote readout of temperature by analyzing spectroscopic properties of phosphor. Although the ratiometric approach is the most popular noncontact temperature sensing technique described in the literature, the reliability and accuracy of the temperature readout are significantly limited by the fact that the ratio of the intensity of the two bands can be modified by the medium in which the phosphor is located.^17,20^

Therefore, an important alternative to the ratiometric technique is the lifetime-based approach.^17,20−24^ In the case of luminescence kinetics, the absorption of the emitted radiation by the medium does not significantly affect the lifetimes of excited states and thus does not modify the calibration curve of the thermometer. To increase the sensitivity of a thermometer based on luminescence kinetics, physical processes are sought that will significantly affect the thermal dynamics of the emission level depopulation in a predictable way. In the case of lanthanide ions, the main process responsible for the thermal depopulation of the excited level is the multiphonon depopulation process. However, the probability of this process increases when the number of phonons required to energetically bridge the emitting level with the next lower-lying level decreases. Unfortunately, this leads to the reduction of the luminescence intensity. Therefore, recently there has been increasing interest in the exploitation of alternative processes, among which phonon-assisted energy transfer provides very promising results.^25−27^ In this case, a luminescent ion characterized by high luminescence intensity provided by a high energy separation between the excited and the ground levels and a codoped ion with a configuration of energy levels that serve as an energy acceptor are desirable.^28^ An ideal pair of ions fulfilling these requirements is Yb^3+^, Nd^3+^ ions, where the distance between the ground ^2^F_7/2_ and excited ^2^F_5/2_ levels of about 10,000 cm^–1^ limits the probability of multiphonon processes and the ^4^F_3/2_ level of Nd^3+^ ions located about 1500 cm^–1^ above ^2^F_5/2_ can be populated after the absorption of one to two host phonons.^29,30^ The probability of this process strongly depends on the temperature. A luminescent thermometer could be developed on the basis of this finding.^25−27^ An additional advantage of using this ion pair is the fact that they operate in the near-infrared spectral range, which can be important for many applications, e.g., biomedicine.^31−33^ To develop thermometers with the desired properties and thermometric parameters, it is important to understand what material parameters and how they affect the thermometric performance of the phosphor. As our previous studies have shown, the chemical composition of the host material significantly affects the thermometric parameters in APO_4_:Nd^3+^,Yb^3+^ (A = Y, Lu, La, Gd).^25^

**Figure 1:**
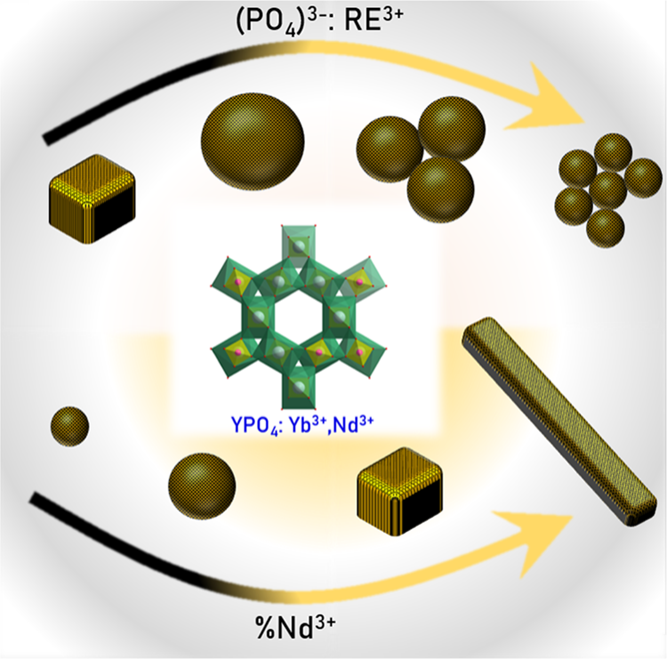
Schematic representation of the influence of the synthesis condition on the morphology of the YPO_4_:Nd^3+^,Yb^3+^ nanocrystals.

In this work, the effect of the synthesis parameters on the morphology of YPO_4_:Nd^3+^,Yb^3+^ nanocrystals and their consequences on the thermometric parameters of the phosphor are analyzed. For this purpose, the solvothermal synthesis method in a mixture of ethanol and oleic acid was used.^34,35^ It was observed that with the increase in the molar concentration of (PO_4_)^3–^ ions relative to RE^3+^ cations, the cubic nanoparticles (for (PO_4_)^3–^:RE^3+^ = 0.5) change their shape to spherical ((PO_4_)^3–^:RE^3+^ = 1). Further decrease in the (PO_4_)^3–^ concentration causes the lowering of the particle size and facilitates their aggregation. On the other hand, with the increasing concentration of Nd^3+^ ions, the morphology of nanocrystals also undergoes a significant modification from spherical nanoparticles, with the shape of nanoparticles changing to cubic and then elongating along one axis to form elongated cuboids (Figure 1).

## 2 Experimental Section

Ytterbium(III) oxide Yb_2_O_3_ (99.99%, Alfa Aesar), europium(III) oxide Eu_2_O_3_ (99.99%, Alfa Aesar), neodymium(III) oxide Nd_2_O_3_ (99.9%, Alfa Aesar), yttrium(III) oxide Y_2_O_3_ (99.99%, Alfa Aesar), sodium phosphate Na_3_PO_4_ (98.0%, Alfa Aesar), oleic acid (90%, Sigma Aldrich), sodium hydroxide (99.8%, POCH S.A., Poland), ethanol (96% pure p.a. POCH S.A. Poland), methanol (POCH S.A., Poland), *n*-hexane (POCH S.A., Poland), and chloroform (POCH, Poland) were used without further purification.

In a typical synthesis in a 50 mL autoclave, 0.6 g of NaOH was dissolved into 5 mL of deionized water under stirring. The rare-earth (RE) oxides were dissolved in hydrochloric acid to obtain chloride salts. The resulting rare-earth salts were adjusted to neutral pH by recrystallizing three times with distilled water. Then, the rare-earth chlorides (0.2 M) were added. Thereafter, 10 mL of ethanol and 10 mL of oleic acid were added under vigorous stirring. After 1 h, 5 mL of NaH_2_PO_4_ aqueous solution (*x* = 0.1, 0.2, 0.5, and 1 M) and 10 mL of ethanol were added to the autoclave after stirring for another 30 min. Then, the autoclave was sealed and heated at 180 °C for 8 h. The solution was cooled to room temperature and the nanoparticles were washed with hexane/ethanol by centrifugation. The final product was redispersed in 5 mL of chloroform.

### 2.1 Methods

Powder diffraction data were obtained using a PANalytical X’Pert Pro diffractometer equipped with an Anton Paar TCU 1000 N temperature control unit using Ni-filtered Cu Kα radiation (*V* = 40 kV, *I* = 30 mA). Transmission electron microscopy (TEM) images were performed with a Philips CM-20 SuperTwin transmission electron microscope operating at 160 kV. A drop of the suspension was put on a copper microscope grid covered with carbon. Before the measurement, the sample was dried and purified in an H_2_/O_2_ plasma cleaner for 1 min. The hydrodynamic size of nanoparticles was determined by dynamic light scattering (DLS) using Malvern ZetaSizer at room temperature in a quartz cuvette using hexane as a dispersant. Measurements were made using histograms representing the number of particles per their average size. The emission spectra and luminescence decay profiles were measured using an FLS1000 fluorescence spectrometer from Edinburgh Instruments equipped with a 450 W Xenon lamp and 808 nm line laser as excitation sources and an R928P side window photomultiplier tube from Hamamatsu as a detector. The temperature of the sample was controlled using a THMS 600 heating–cooling stage from Linkam.

## 3 Results and Discussion

### 3.1 Structural and Morphological Characterization

**Figure 2:**
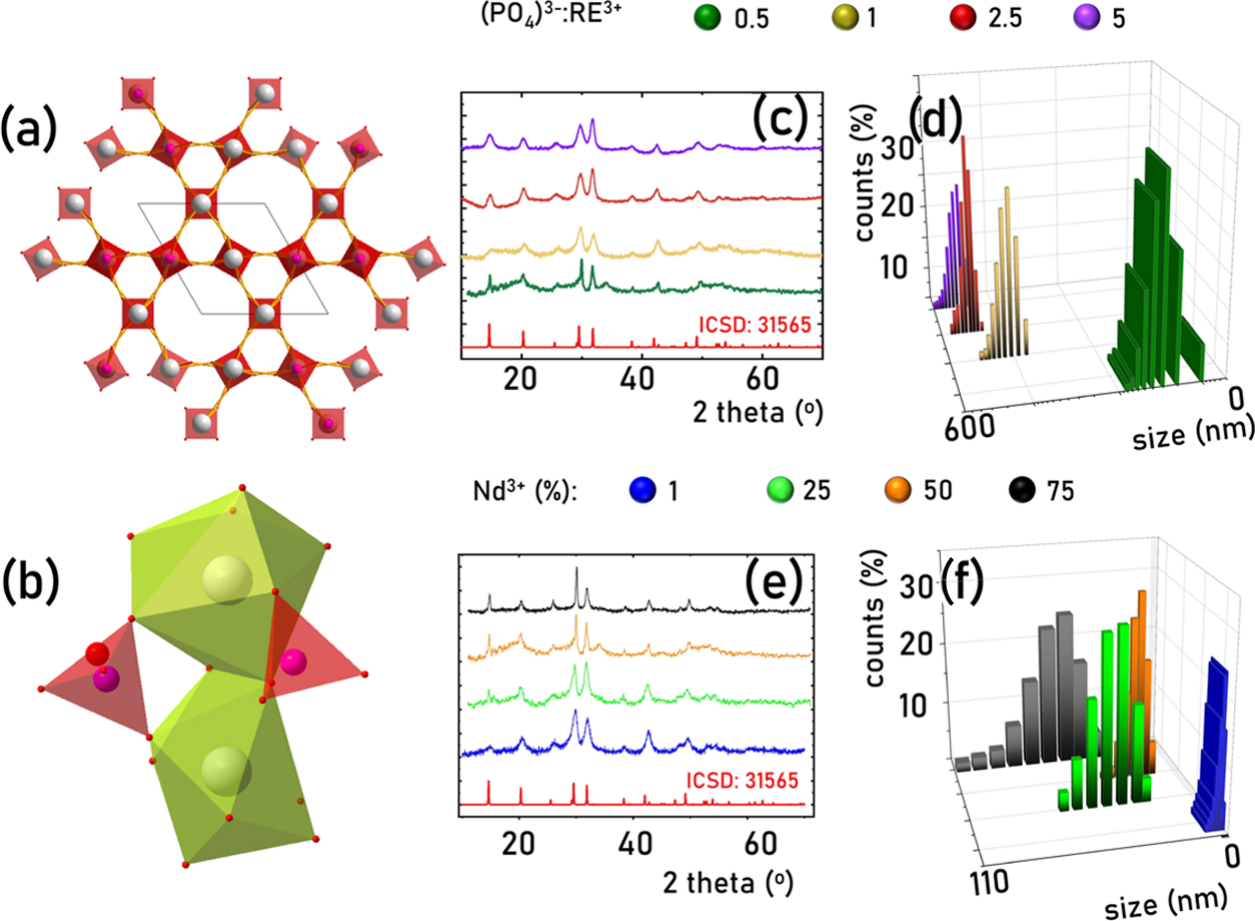
Visualization of the hexagonal structure of YPO_4_ (a) and the polyhedra of Y^3+^ ions (b). (c) XRD patterns of YPO_4_:10% Yb^3+^, 50% Nd^3+^ synthesized with different RE^3+^:(PO_4_)^3–^ ratios and (d) the corresponding particle size distribution obtained from DLS measurements. (e) XRD patterns for YPO_4_:10% Yb^3+^ and different concentrations of Nd^3+^ ions and (f) the corresponding particle size distribution obtained from DLS measurements.

Depending on the synthesis condition, the YPO_4_ crystallizes in the tetragonal or hexagonal structures.^36−38^ However, a tetragonal structure is usually observed when higher annealing temperatures are applied, whereas in the case of the as-prepared nanocrystals, a typical hexagonal structure of the *P*_6_222 space group can be found (Figure 2a). In this host material, the Y^3+^ ions are coordinated by eight O^2–^ ions, and this dodecahedral site can be successfully substituted by lanthanide dopant ions (Figure 2b).^36−38^ Despite many factors such as pH, reaction time, and solvent volume ratio, the (PO_4_)^3–^:RE^3+^ ratio can most significantly influence the nanocrystal growth process. Therefore, the first aspect in the optimization of the synthesis conditions of YPO_4_:Nd^3+^,Yb^3+^ nanocrystals is to find a proper ratio between phosphate ions (PO_4_)^3–^ and rare-earth ions (RE). It is found that even for the nonstoichiometric ratio between ions, pure phased nanocrystals can be obtained (Figure 2c). However, the comparison of the X-ray diffraction (XRD) patterns of the nanocrystals synthesized using different ionic ratios does not reveal any phase impurities. One can observe that for samples with different Nd^3+^ concentrations exhibit a slight shift in the reflections toward lower 2θ angles (e.g., from 20.48 to 20.17° for the most intense reflection). This is due to the expansion of the crystallographic cell unit associated with the ionic difference between Nd^3+^ and Y^3+^ ions, which may also be associated with the preferred orientation of the particles or the preferred growth direction of the nanoparticles. The average particle size determined using the DLS technique indicates that when the (PO_4_)^3–^:RE^3+^ ratio is below 1, the nanoparticles of average size around 26 nm are obtained (Figure 2d). When the stoichiometric amount of (PO_4_)^3–^ ions is applied, an increase in the particle size to 107 nm is found. A further decrease in the molar amount of the phosphate ions results in an increase in size to 250 nm and 270 nm for the ratios 2 and 5, respectively. However, as is well known, the results obtained from the DLS technique for nonspherical and highly aggregated particles can be misleading. This is particularly important when a PDI close to 1 is obtained. However, in the case of the examined materials with nonaggregated particle form, the PDI values range from 0.002 to 0.310; therefore, the quality of the measurements is satisfactory. The analysis of the TEM images (Figure S1) reveals that for a low (PO_4_)^3–^:RE^3+^ ratio of 0.5, the cubic nanoparticles can be obtained. An increase in the stoichiometric ratio causes the crystallization of relatively large spherical particles. Further increase in the (PO_4_)^3–^:RE^3+^ ratio reduces the size of the particles and facilitates their aggregation. Since the small particle size and nonaggregation are optimized for (PO_4_)^3–^:RE^3+^ = 0.5, this molar ratio is applied in further syntheses. In the second step of the analysis, the influence of the Nd^3+^ concentration on the structural and morphological properties of the YPO_4_:Nd^3+^,Yb^3+^ nanocrystals is investigated. It is found that even for 75% Nd^3+^, the hexagonal structure remains (Figure 2e). However, the morphology differs significantly when the Nd^3+^ concentration is increased. In the case of as low as 1% Nd^3+^, the spherical particles are achieved with a diameter of around 14 nm, and an increase in Nd^3+^ to 25% results in the enlargement of their size to around 60 nm. Surprisingly, a further increase in the Nd^3+^ concentration enables us to achieve a cube with a size of 40 nm (50% Nd^3+^) and even rodlike particles with a length of 120 nm and a width of 15 nm (75% Nd^3+^). As can be clearly seen, the results obtained using the DLS technique are not reliable in this case (Figure 2f).

**Figure 3:**
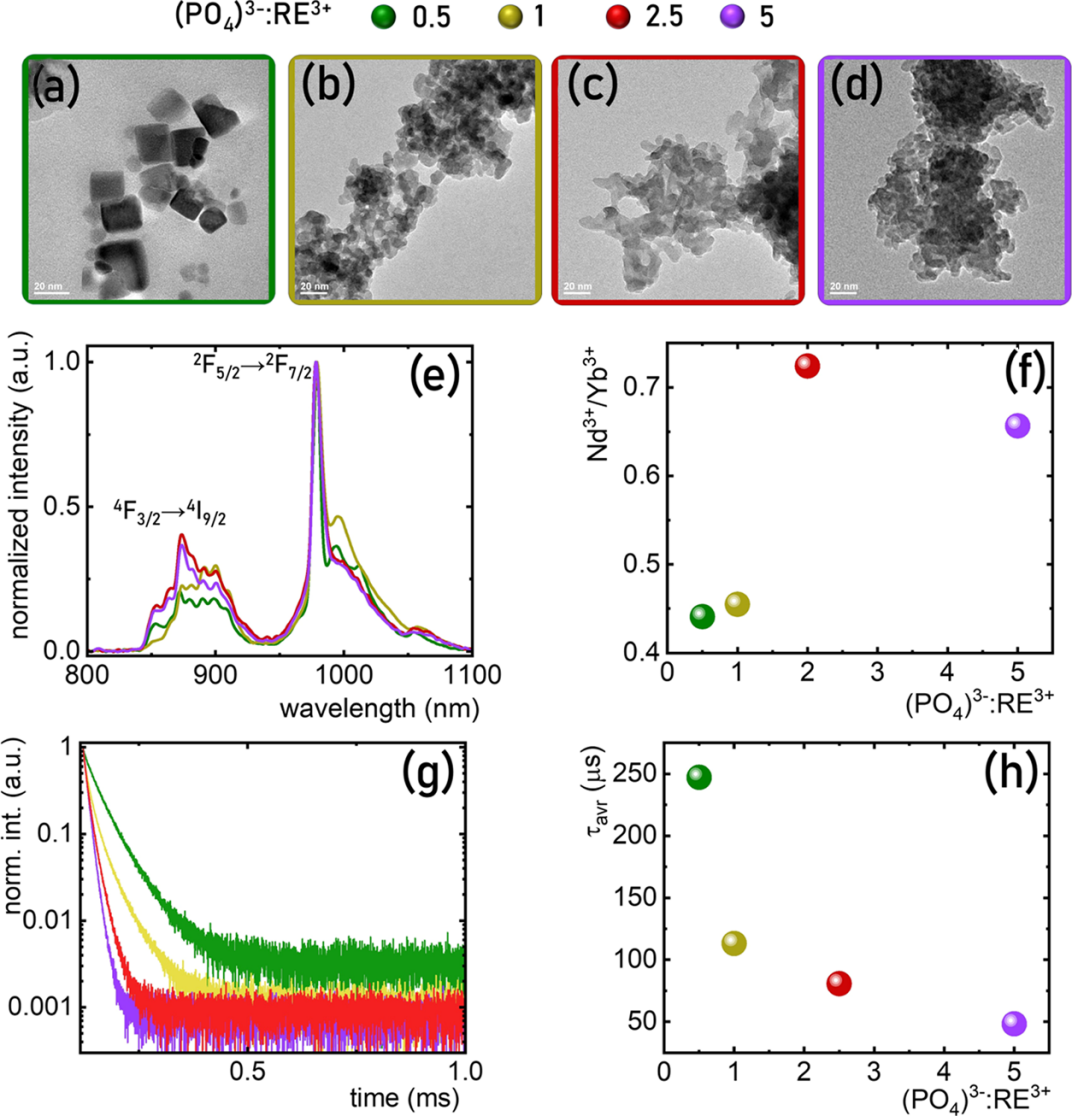
Representative TEM images of the YPO_4_:10% Yb^3+^, 50% Nd^3+^ nanoparticles synthesized using different (PO_4_)^3–^:RE^3+^ ratios of 0.5 (a), 1 (b), 2.5 (c), and 5 (d). (e) Comparison of the normalized emission spectra of YPO_4_:10% Yb^3+^, 50% Nd^3+^ nanoparticles measured at 77 K upon λ_exc_ = 808 nm, (f) emission intensity ratio of Nd^3+^ to Yb^3+^ ions, (g) luminescence decay profile of Yb^3+^ ions, and (h) the corresponding τ_avr_ of the ^2^F_5/2_ state of Yb^3+^ ions as a function of (PO_4_)^3–^:RE^3+^ ratio.

The luminescent properties of the Nd^3+^ and Yb^3+^ ions are very well known.^39−41^ Therefore, only a brief description is given here. Upon 808 nm excitation, the excited state ^4^F_5/2_ of the Nd^3+^ ions can be populated, followed by the fast nonradiative processes causing the population of the emitting ^4^F_3/2_ state of the Nd^3+^. Radiative depopulation of the ^4^F_3/2_ state leads to the occurrence of the three emission bands at around 880, 1060, and 1350 nm that can be assigned to the ^4^F_3/2_ → ^4^I_9/2_, ^4^F_3/2_ → ^4^I_11/2_, and ^4^F_3/2_ → ^4^I_13/2_ transitions, respectively. In the presence of the Yb^3+^ codopant, the ^4^F_3/2_ state of the Nd^3+^ ions can be also depopulated by the energy transfer with the emission of the phonon to the ^2^F_5/2_ state of the Yb^3+^ ions. The high energy separation between the ^2^F_5/2_ state and the ^2^F_7/2_ ground state (around 10,000 cm^–1^) prevents efficient depopulation of the excited state of Yb^3+^ by multiphonon relaxation. However, an increase in temperature results in the growing probability of the back energy transfer to the Nd^3+^ with the absorption of phonons, thus shortening its lifetime. This preserves high thermal variability of the lifetime of the Yb^3+^ ions and thus is beneficial from the thermometric perspective. However, in the case of the nanocrystals also, the surface effect significantly affects the luminescent properties of the dopant ions.^42−45^ In the case of the small particles, the energy diffusion among excited states of Yb^3+^ ions to the surface defects becomes a very efficient channel for the quenching of the luminescence. Thus, the smaller the particle, the shorter the lifetime that is expected in this case. As can be seen, the applied different RE^3+^:(PO_4_)^3–^ ratios affect the morphology of the YPO_4_:10% Yb^3+^, 50% Nd^3+^ significantly (Figure 3a–d). Hence, changes in the spectroscopic properties of these particles are expected. The comparison of the low-temperature emission spectra of the YPO_4_:10% Yb^3+^, 50% Nd^3+^ reveals that some changes can be found in the shape of the ^4^F_3/2_ → ^4^I_9/2_ emission band when the RE^3+^:(PO_4_)^3–^ is changed (Figure 3e). The smaller the particle size, the higher intensity of R_1_ → Z_1_ and R_2_ → Z_1_ electronic transitions between particular Stark components of excited and ground states. This effect can be explained in terms of the energy reabsorption between Nd^3+^ ions.^46^ When the concentration of Nd^3+^ is high, the light emitted by one of the ions can be absorbed by the other before being emitted from the nanoparticle. The probability of energy reabsorption is the highest for the resonant lines since the population of the Z_1_ Stark sublevel of the ^4^I_9/2_ state is the highest. The larger the size of the particle, the higher the probability of this process because the longer distance photon needs to travel before it leaves the nanoparticle. Therefore, for smaller nanoparticles, more intense R_1_ → Z_1_ and R_2_ → Z_1_ emission lines can be found by comparing larger counterparts.

Also, the emission intensity ratio of Nd^3+^ to Yb^3+^ ions is modified by the size of the particles (Figure 3f). The reduction of the particle size leads to a monotonic increase in the Nd^3+^-to-Yb^3+^ ratio. This effect can be caused by either the reduction of the Yb^3+^ emission intensity or a growing emission intensity of Nd^3+^. Since the spectral position of the emission bands is independent of the RE^3+^:(PO_4_)^3–^ ratio, the energy-level configuration of Yb^3+^ and Nd^3+^ remains unchanged. Hence, the probability of the interionic energy transfer is expected to be independent of the particle size in this case. Therefore, the observed phenomenon can be explained in terms of two effects. First of them is the light-induced heating of the particles and associated with this more promoted transfer of the electrons from Yb^3+^ to Nd^3+^. The efficiency of heat dissipation in strongly dimensionally constrained objects like nanoparticles is strongly limited. Hence, light-induced heating is more efficient for smaller objects. On the other hand, the reduction of the particle size causes an enhancement of the probability of the quenching of the excited state of Yb^3+^ ions via surface-related quenching processes. Undoubtedly, the change in Yb^3+^ emission intensity affects the ratio of luminescence intensity of Nd^3+^ to Yb^3+^ ions, which is confirmed by the analysis of luminescence kinetics of Yb^3+^ ions (Figure 3g). It is clearly seen that with the increasing RE^3+^:(PO_4_)^3–^ concentration, the average lifetime (τ_avr_, eqs S1 and S2) of the ^2^F_7/2_ level is monotonically shortened from τ_avr_ = 250 μs for RE = 0.5 to τ_avr_ = 48 μs for RE = 5.

To verify the influence of the particle size on the luminescence thermometer parameters based on the lifetime of the ^2^F_7/2_ level of Yb^3+^ ions, the luminescence kinetics was measured as a function of temperature and the average lifetimes were determined (Figures S2 and 4a). As shown in the presented analysis for RE^3+^:(PO_4_)^3–^ = 0.5, τ_avr_ shortens significantly above 200 K up to about 350 K, above which the rate of observed changes slows down. An increase in the RE^3+^:(PO_4_)^3–^ ratio, as shown earlier, reduces the value of τ_avr_ and an increase in temperature causes an additional shortening of its value but at a much lower rate than is the case for larger nanoparticles. This effect can be explained by the increased role of surface processes. As it is well known, in the case of materials doped with Yb^3+^ ions, the energy diffusion across excited ^2^F_5/2_ states is very efficient.^32,47,48^ For nanomaterials, such a process due to the relatively short ion–surface distance very often leads to energy quenching at the nanocrystal surface through interactions with ligands and functional groups on the surface or on surface structural defects.^42−45^ The energy diffusion is a resonant process and its probability should not depend on the temperature, so it effectively shortens the lifetime of Yb^3+^ ions when it becomes more efficient by reducing the nanoparticle. From a thermometric perspective, this process can be seen as competing with the energy transfer process between Yb^3+^ and Nd^3+^; whereas the latter favors increasing the sensitivity of the thermometer, the former suppresses the thermal dynamics of the lifetime changes. For small nanoparticles, it is so efficient that further temperature-induced shortening of τ_avr_ values is less evident. The rate of temperature-induced shortening of the Yb^3+^ ion lifetime can be quantified by the absolute sensitivity (*S*_A_) of the luminescence thermometer determined by the following formula^14^
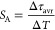
1where Δτ_avr_ represents the change in τ_avr_ corresponding to the change in temperature by Δ*T*. As expected, the highest *S*_A_ values were obtained for the largest nanoparticles because the longest τ_avr_ values were found for these nanoparticles. The maximum value of *S*_A_ = 1.36 μs/K was obtained at 275 K. Decreasing the size of nanoparticles resulted in a successive decrease in the *S*_A_ value. For the smallest nanoparticles (RE^3+^:(PO_4_)^3–^ ratio = 5), the maximum *S*_A_ = 0.06 μs/K at 220 K. For a further quantitative comparative analysis, the relative sensitivity was also determined according to the equation^14^
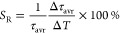
2In this case, the highest value of *S*_R_ = 0.9%/K was obtained at 300 K for YPO_4_:10% Yb^3+^,50% Nd^3+^ synthesized using RE^3+^:(PO_4_)^3–^ = 0.5 (Figure 5c). It was worth noting that the temperature at which the maximum sensitivity was obtained for individual samples depended on the size of the nanoparticles and a general trend could be observed that it decreased with the reduction of the size of the luminescent thermometer. The maximum values of *S*_R_ decreased monotonically with the RE^3+^:(PO_4_)^3–^ (Figure 5d). The analysis carried out allowed us to conclude that to increase the sensitivity of the luminescence thermometer based on luminescence lifetimes, it was advantageous to use nanoparticles of a larger size. It should be noted here that the hexagonal structure of the YPO_4_ was stabilized by the crystallographic OH groups that could affect the population of the excited state of the ^2^F_5/2_ state of Yb^3+^ ions. Hence, the removal of the OH groups at higher temperatures may influence the τavr. However, according to the TG-DSC analysis shown by Li et al. in the hexagonal YPO_4_, this removal was observed around 470 K.^49^ This temperature exceeded the analyzed temperature range in this study.

**Figure 4:**
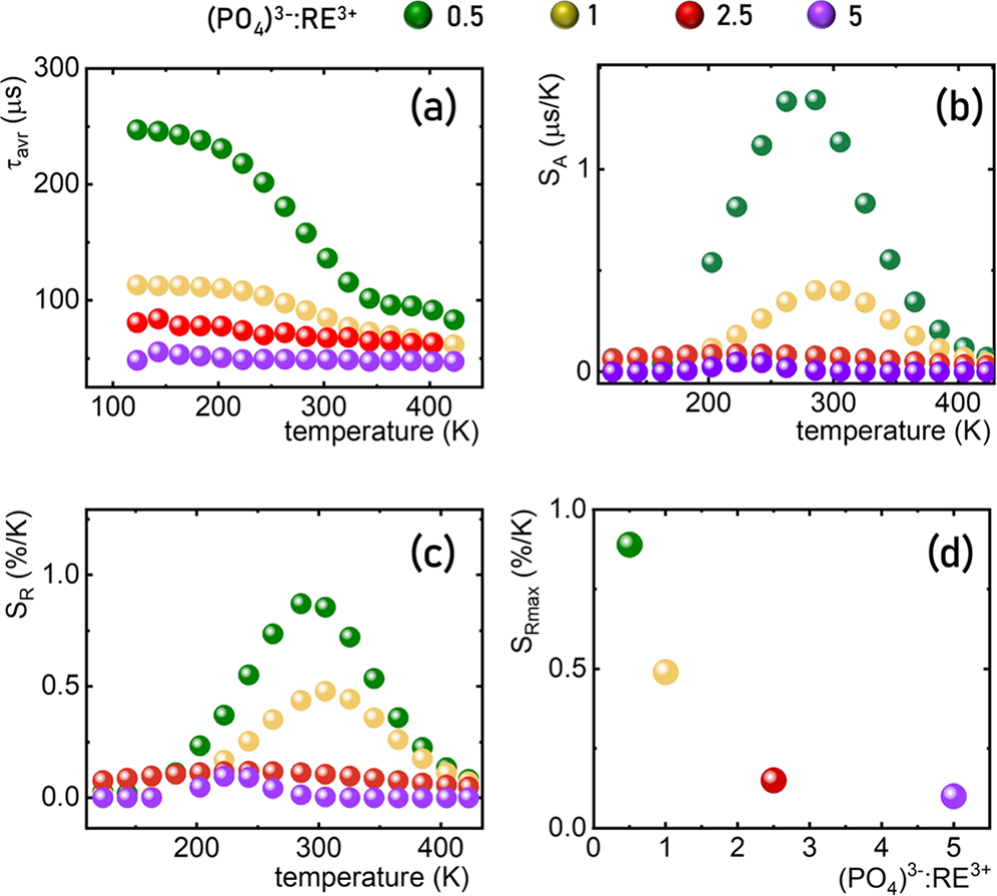
(a) Thermal dependence of the τ_avr_ of Yb^3+^ ions obtained for different RE^3+^:(PO_4_)^3–^ ratios. The corresponding *S*_A_ (b) and *S*_R_ (c). (d) Influence of the RE^3+^:(PO_4_)^3–^ on the maximal *S*_R_ (d).

**Figure 5:**
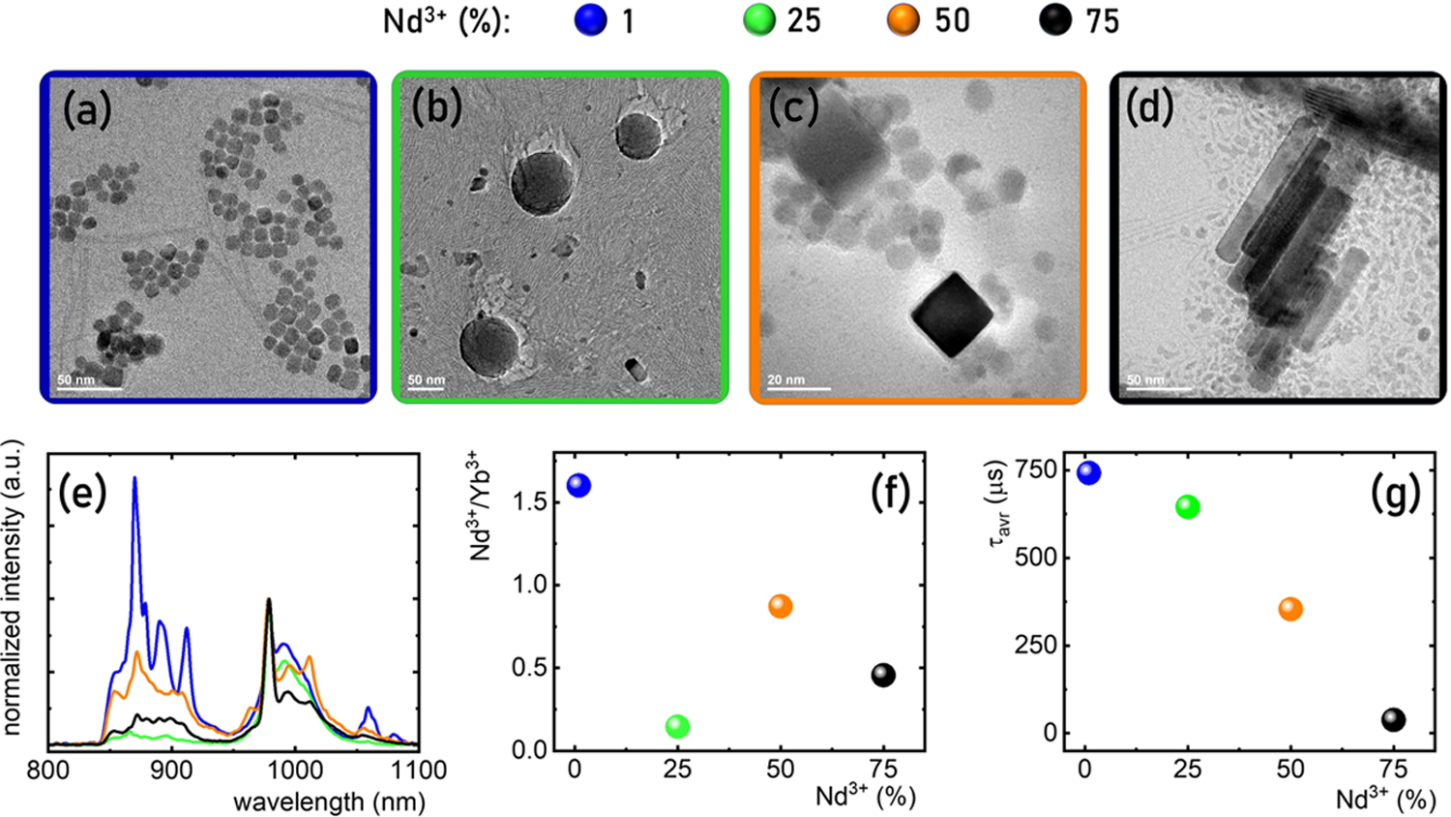
Representative TEM images for YPO_4_:10% Yb^3+^, % Nd^3+^ nanoparticles with different Nd^3+^ concentrations: 1% Nd^3+^ (a), 25% Nd^3+^ (b), 50% Nd^3+^ (c), and 75% Nd^3+^ (d). (e) Comparison of the low-temperature emission spectra upon λ_exc_ = 808 nm for different Nd^3+^ concentrations. (f) Nd^3+^ to Yb^3+^ emission intensity ratio as a function of Nd^3+^ concentration. (g) Influence of the Nd^3+^ on the τ_avr_ of ^2^F_5/2_ state of Yb^3+^ ions.

As indicated by the above studies, the nanocrystal morphology had a significant effect on the thermometric performance of a luminescent thermometer based on the luminescence decay times from the ^2^F_5/2_ level. Taking into account that the phonon-assisted energy transfer to the neighboring Nd^3+^ ion was responsible for the thermal lifetime shortening of this level, one could expect that increasing the Nd^3+^ ion concentration would facilitate this energy transfer by shortening the average Nd^3+^–Yb^3+^ distance. Therefore, the thermometric properties of such a thermometer were expected to depend significantly on the concentration of Nd^3+^ ions. On the other hand, it was known that REPO_4_ phosphates with large RE ionic radii (such as Nd^3+^) preferentially crystallized in the monoclinic structure and revealed an increased tendency to form elongated rodlike structures.^50^ Therefore, it was important to verify how the concentration of Nd^3+^ ions affected the morphology and spectroscopic properties of the obtained phosphors. As mentioned above and illustrated by representative TEM images, the increase in the concentration of Nd^3+^ ions significantly modified the morphology of the sample. For a concentration of 1% Nd^3+^, small, spherical, and nonaggregated nanoparticles were observed (Figure 5a). An increase in the Nd^3+^ concentration to 25% enlarged the size of the nanoparticles while maintaining their shape (Figure 5b). However, the increase in the Nd^3+^ ion concentration to 50% caused a slight decrease in the size of the nanoparticles. Additionally, apart from spherical particles, cubic ones could also be observed (Figure 5c). Further increase in the concentration of Nd^3+^ ions resulted in the elongation of the nanoparticles’ shape by their growth along the c-axis (Figure 5d).^50^ Importantly, even for such high concentrations of Nd^3+^ ions, the hexagonal structure was maintained (Figure 2). A comparison of the luminescence spectra of YPO_4_:10% Yb^3+^,% Nd^3+^ with different concentrations of Nd^3+^ ions revealed that this change in concentration caused the reduction of the luminescence intensity of the ^4^F_3/2_ → ^4^I_9/2_ band of Nd^3+^ ions relative to the ^2^F_5/2_ → ^2^F_7/2_ band of Yb^3+^ ions. This was mainly due to an increase in the quenching of the excited level of ^4^F_3/2_ through the {^4^F_3/2_, ^4^I_9/2_} ↔ {^4^I_15/2_, ^4^I_15/2_} cross-relaxation process. Additionally, the shape of the emission bands changed slightly, revealing a larger number of Stark components for both Nd^3+^ and Yb^3+^ bands. This effect suggested a decrease in the local symmetry of the lanthanide ions as the concentration of Nd^3+^ ions increased associated with the transition to the monoclinic NdPO_4_ structure. Considering the fact that XRD patterns confirmed the presence of only hexagonal structure, the change in the shape of the emission spectrum might indicate the local character of the mentioned changes. Assuming that the observed symmetry changes occurred only in the inner part of the nanocrystal and the hexagonal phase was stabilized mainly in the surface part of the nanocrystal, one could expect that the luminescence spectra would be dominated by the contribution from ions localized in the reduced symmetry. However, this was a speculative hypothesis and required more in-depth investigation in the future. The ratio of the luminescence intensity of Nd^3+^ ions to Yb^3+^ ions decreased almost linearly with increasing Nd^3+^ ion concentration (Figure 5f). As expected, the lifetime of the ^2^F_5/2_ level shortened with the increasing Nd^3+^ concentration, and the monotonic nature of this shortening with no deviation around between 25% Nd^3+^ and 75% Nd^3+^ where significant changes in nanoparticle shape were observed indicated that the dominant factor causing the shortening was the effect of shortening the average distance between interacting ions.

**Figure 6:**
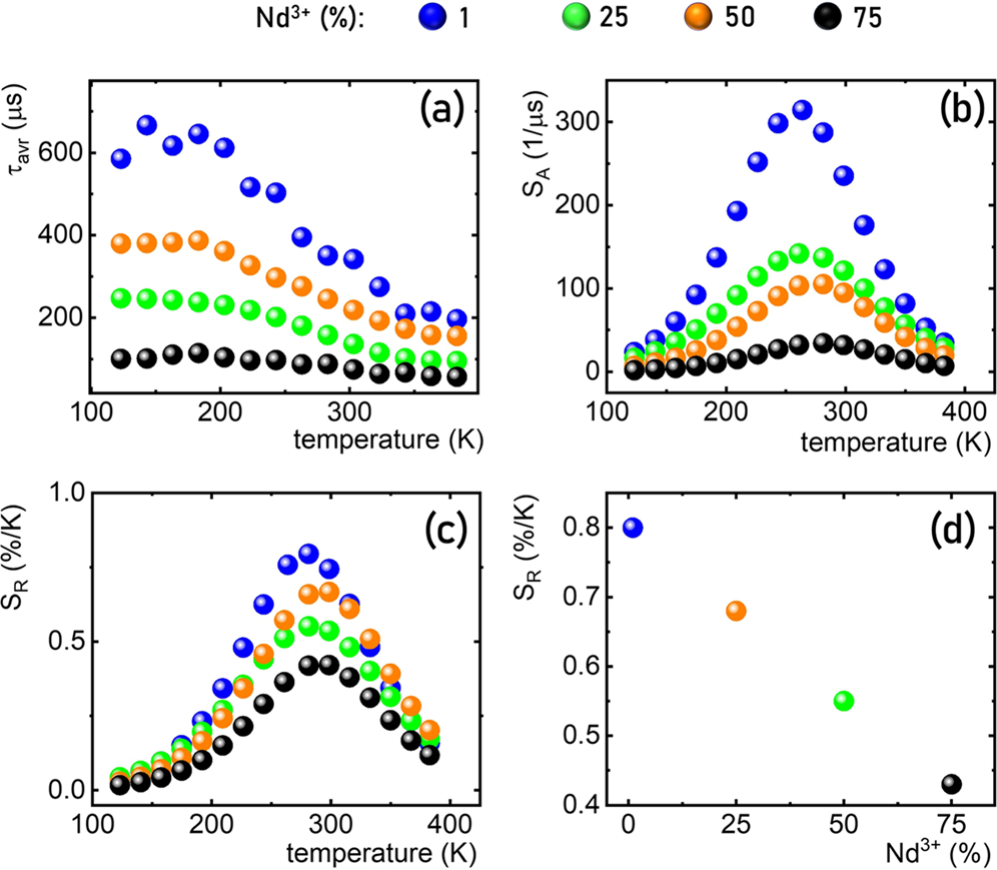
Thermal dependence of the τ_avr_ of ^2^F_5/2_ state of Yb^3+^ ions in YPO_4_:10% Yb^3+^, Nd^3+^ for different concentrations of Nd^3+^ ions (a); *S*_A_ (b) and *S*_R_ (c); and influence of the Nd^3+^ concentration on the maximal achieved *S*_R_ (d).

As can be seen, regardless of the Nd^3+^ ion concentration, τ_avr_ shortens above 190 K. However, the rate of this shortening clearly depends on the dopant concentration (Figures 6a and S3). As indicated above, the high efficiency of the phonon energy transfer process associated with an increase in dopant ion concentration results in a shortening of τ_avr_. Therefore, the additional lifetime shortening associated with phonon absorption is less efficient (Figure 6a). Hence, the highest absolute sensitivities and the relative sensitivities are observed for low dopant concentrations. Importantly, a linear decrease in the maximum relative sensitivity is observed with increasing concentration. This directly demonstrates that although increasing the Nd^3+^ concentration causes a change in sample morphology (which should result in improved thermometric performance as the nanoparticle size increases), the increased probability of phonon-assisted energy transfer plays a dominant role in this case. The high repeatability of the τ_avr_ within several heating–cooling cycles (Figure S5) and low-temperature determination uncertainty (δ*T* < 0.1 K) confirms the high applicative potential of the presented luminescence thermometers.

## 4 Conclusions

The solvothermal synthesis parameters of YPO_4_:Nd^3+^,Yb^3+^ nanocrystals significantly affect the morphology of the final product. It has been shown that using low RE^3+^:(PO_4_)^3–^ concentrations can deliver cubic and well-separated nanocrystals while increasing the RE^3+^:(PO_4_ )^3–^ ratio results in a decrease in the size of nanoparticles, changing their shape to spherical and increasing the degree of aggregation. This significantly affects not only the spectroscopic properties of YPO_4_:Yb^3+^,Nd^3+^ but also the thermometric performance of the thermometer based on the luminescence kinetics of the ^2^F_5/2_ level of Yb^3+^ ions. The decrease in nanoparticle size increases the role of surface quenching processes in the luminescence of Yb^3+^ ions manifested in the shortening of the lifetime of the ^2^F_5/2_ level and decrease in the emission intensity of the ^2^F_5/2_ → ^2^F_7/2_ band with respect to the ^4^F_3/2_ → ^4^I_9/2_ band of Nd^3+^ ions. The surface luminescence quenching processes competing with the energy transfer process involving Yb^3+^ → Nd^3+^ phonons decrease the absolute and relative sensitivity of the luminescence thermometer. An increase in the concentration of Nd^3+^ ions changes the shape of nanoparticles from spherical to cubic (50% Nd^3+^) or even rodlike (75% Nd^3+^). Cross-relaxation-activated processes decrease the luminescence intensity of the Nd^3+^ band relative to the Yb^3+^ band with the increasing Nd^3+^ concentration. Interestingly, the shape of the luminescence spectra recorded for Nd^3+^ concentrations above 50% suggests that a monoclinic structural phase is present in the samples despite the fact that the XRD falls indicate the presence of only hexagonal structure. Considering the linear decrease in the maximum relative sensitivity of the luminescent thermometer with increasing Nd^3+^ ion concentration, it can be concluded that the concentration of Nd^3+^ ions has a dominant influence on the thermometric parameters of this type of thermometer. The presented studies indicate the important role of nanocrystal morphology on thermometric performance, which is often not considered, or not analyzed.

## Supporting Information Available

Additional TEM images and luminescence decay profiles measured at different temperatures (PDF)
