# Supplementary material for: Influence of the Synthesis Conditions on the Morphology and Thermometric Properties of the Lifetime-Based Luminescent Thermometers in YPO4:Yb3+,Nd3+ Nanocrystals
Source: ACS Omega. 2022 Aug 24;7(35):31466–73. doi: 10.1021/acsomega.2c03990 (PMC9453944; doi:10.1021/acsomega.2c03990)
Supplement: Supplementary file 1 — ao2c03990_si_001.pdf [file ao2c03990_si_001.pdf]

Supporting Information

The Influence of the Synthesis Conditions on the Morphology and Thermometric Properties of the Lifetime-based Luminescent Thermometers in $YPO_4:Yb^{3+},Nd^{3+}$ Nanocrystals

Kamila Maciejewska[1], Lukasz Marciniak[1*]

[1]Institute of Low Temperature and Structure Research, Polish Academy of Sciences, Okólna 2, 50-422 Wroclaw, Poland

l.marciniak@intibs.pl

Keywords: nanocrystals, shape, luminescence thermometry, YPO_4, lifetime approach

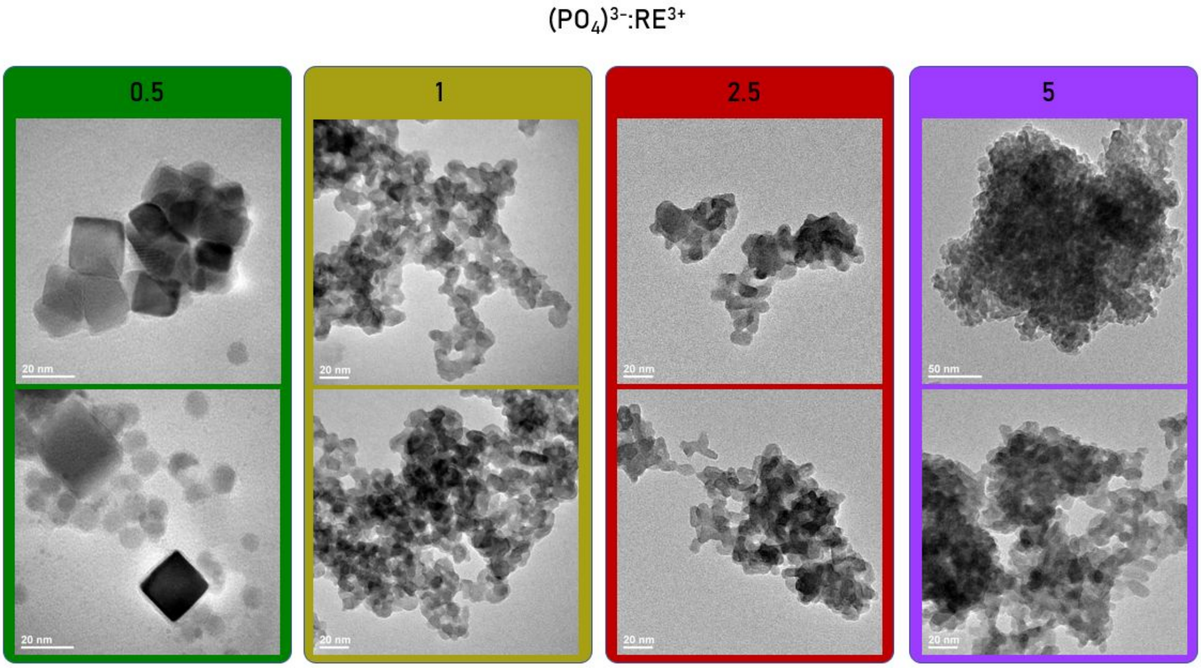

Figure S1. The TEM images of the YPO_4:10%Yb^{3+}, 50%Nd^{3+} nanoparticles obtained with different molar ratio $PO_4^{3-}:RE^{3+}$.

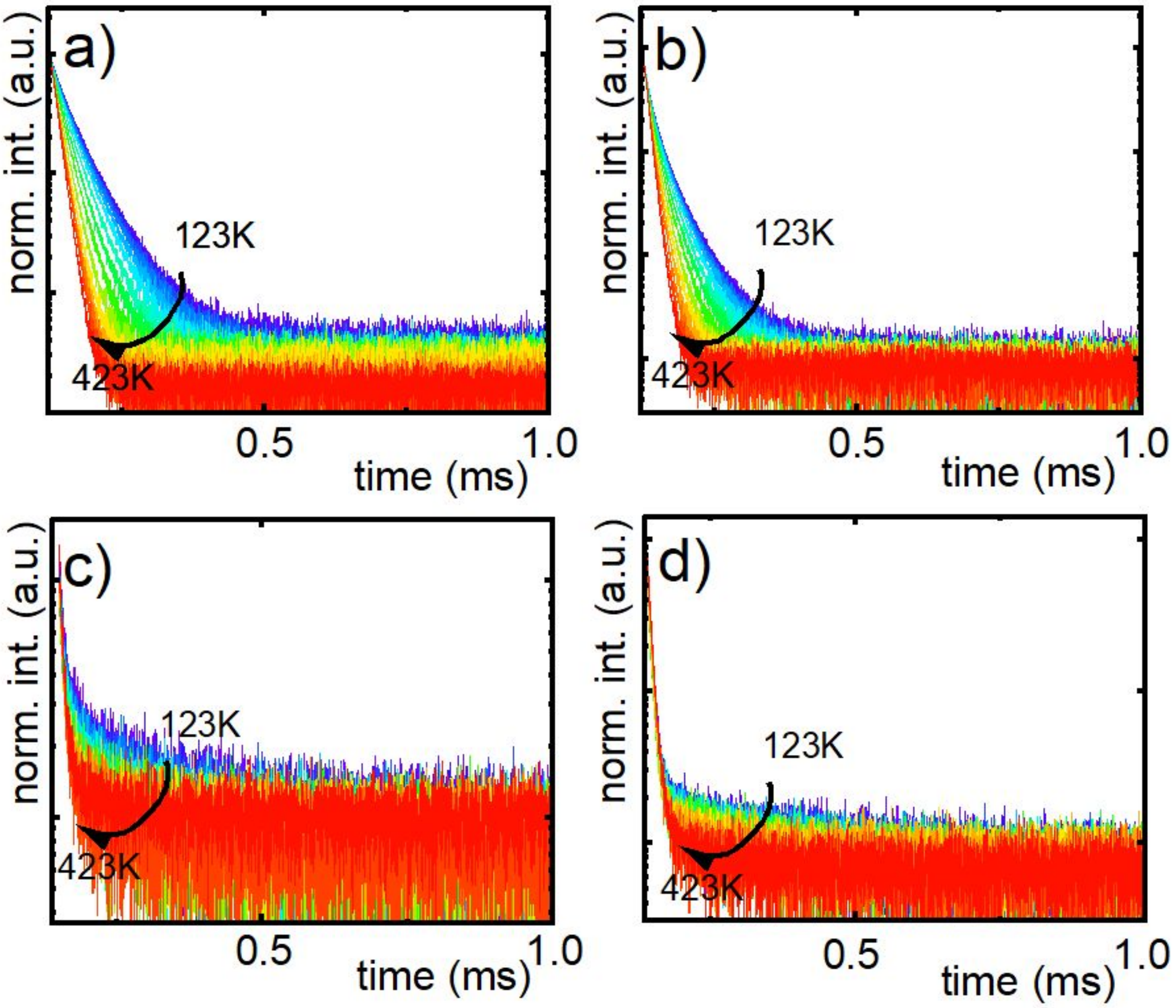

Figure S2. The luminescence decay profiles of Yb^{3+} ions in YPO_4:10%Yb^{3+}, x%$Nd^{3+}$for different $(PO_4)^{3-}$: RE^{3+} ratio: 0.5-a); 1-b); 2.5-c) and 5-d).

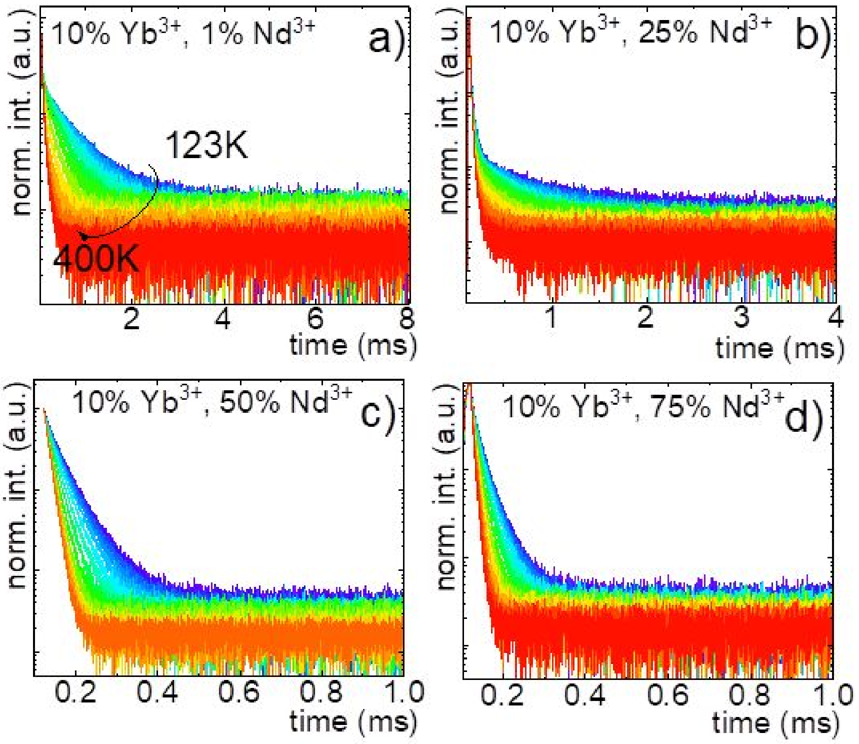

Figure S3. The luminescence decay profiles of Yb^{3+} ions in YPO_4:10%Yb^{3+}, x%Nd^{3+}, where x=1 a); 25 b); 50 c); 75 d)

The luminescence decay profiles were fitted using bi-exponential:

$$y = y_0 + A_1 \cdot \exp(-\frac{x}{t_1}) + A_2 \cdot \exp(-\frac{x}{t_2}) \tag{S1}$$

where y_0 is the initial emission intensity, A_1 and A_2 are the pre-exponential parameters, τ_1 and τ_2 are the decay parameters. Because the luminescence decays were not single exponential, therefore average lifetime (τ_{avr}) was determined using the following equations:

$$\tau_{avr} = \frac{A_1\tau_1^2 + A_2\tau_2^2}{A_1\tau_1 + A_2\tau_2} \tag{S2}$$

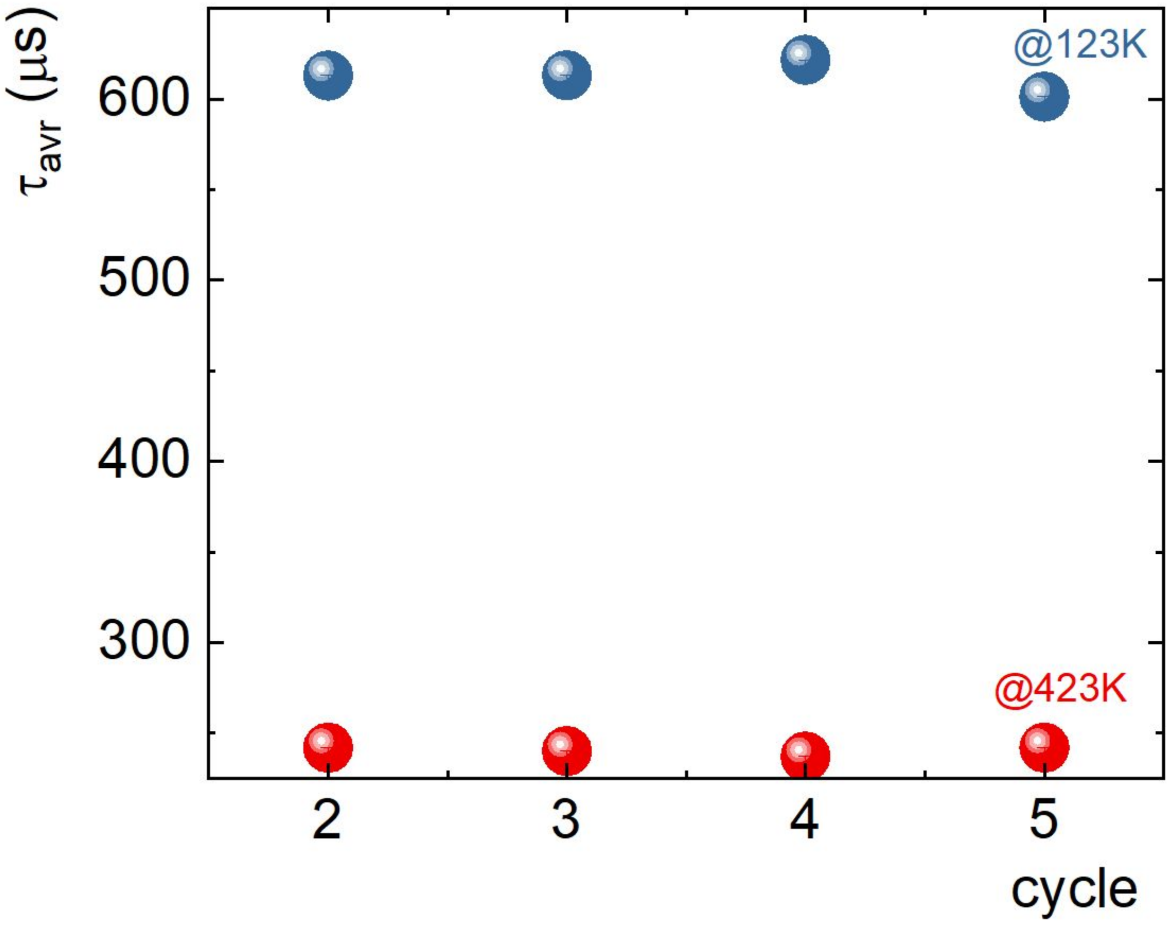

Figure S4. The τ_{avr} measured within a 5 heating-cooling cycles for YPO_4:10Yb^{3+}, 1%Nd^{3+} nanocrystals.

Table S1. Fitting parameter of the luminescence decay profile measured at different temperatures using eq. S1.

T [K]	123	143	163	183	203	223	243	263	283	303	323	343	363	383	403	423
YPO_4:10%Yb^{3+}, 50%Nd^{3+} RE^{3+}:$(PO_4)^{3-}$ ratio =0.5																
y_0	0.00367	0.00359	0.00376	0.00337	0.00336	0.00287	0.00295	0.00275	0.00269	0.00245	0.00269	0.00255	0.00192	0.00168	0.00148	0.00165
A_1	9.68385	1188.121	1236.562	11.2187	11.63516	14.60387	5658.456	29457.3260	72.38999	137936.513	413.16637	1162797.137	41034.0800	0.09689	5086.392	19785.1
A_2	1712.495	9.40881	9.8658	2335.44	1641.6729	2591.253	20.64701	36.36	139551.727	151.46955	1106236.672	1155.12267	742.87951	7679.20894	5107.044	5813.40
τ_1	0.03114	0.01037	0.0103	0.02982	0.02911	0.02717	0.00858	0.00726	0.01839	0.00638	0.01333	0.00547	0.00743	0.03056	0.00916	0.00833
τ_2	0.00983	0.03143	0.0309	0.00949	0.00991	0.00937	0.02472	0.02148	0.00635	0.01584	0.0055	0.01152	0.01161	0.00943	0.00916	0.00833
R^2	0.991	0.99	0.995	0.991	0.993	0.99	0.991	0.992	0.99	0.981	0.995	0.992	0.994	0.991	0.992	0.986
YPO_4:10%Yb^{3+}, 50%Nd^{3+} RE^{3+}:$(PO_4)^{3-}$ ratio =1																
y_0	0.00138	0.0013	0.00126	0.00114	0.00115	0.00109	8.696E-4	9.99482E-4	0.00104	8.53225E-4	9.4171E-4	0.00102	8.38324E-4	8.97326E-4	3.089E-4	7.625E-4
A_1	780535.0	697065.7	15.76757	630132	18.14129	1014653	39.68584	2335514.17	4051797.57	3788743.63	6558059.248	1181.10746	3812378.51	358194.9818	1625.647	707808
A_2	15.91205	15.28663	637451.5	16.2317	635789.40	23.87219	374.5052	55.09105	102.53227	160.7345	351.41748	8610222.878	2305.10473	26.06771	190.5089	836292
τ_1	0.00602	0.00607	0.02188	0.00611	0.02113	0.0059	0.01927	0.00557	0.00537	0.0054	0.00523	0.01056	0.00542	0.00662	0.0034	0.00596
τ_2	0.02193	0.02206	0.00611	0.02169	0.0061	0.02004	0.00245	0.01692	0.01514	0.01389	0.01226	0.00513	0.00965	1.17536E-4	0.01428	0.00596
R^2	0.992	0.99	0.992	0.991	0.995	0.989	0.99	0.991	0.99	0.992	0.994	0.992	0.991	0.99	0.98	0.98
YPO_4:10%Yb^{3+}, 50%Nd^{3+} RE^{3+}:$(PO_4)^{3-}$ ratio =2.5																
y_0	9.835E-4	8.544E-6	1116407	443533	0.00807	1.706E-4	0.00651	3.94337E-4	0.12547	0.00423	123.89367	2.61886	7.96996	0.26901	0.00559	1.182E-4
A_1	8.805E-4	8.482E-6	2165349	818044	0.00781	1.516E-4	0.00548	3.20578E-4	0.13336	0.00462	128.10855	2.4884	7.49871	0.25997	0.00541	1.050E-4
A_2	7.239E-4	6.686E-6	1.684E7	788213	0.00701	1.520E-4	0.00688	5.21488E-4	0.10474	0.00381	142.58029	3.09005	9.54761	0.34718	0.00486	1.053E-4
τ_1	6.727E-4	2.353E-6	0.19003	6.7E47	0.03882	3.112E46	0.02643	4.456E133	7.65686E-4	6.5817E129	25.75667	2.0649E49	1306.01868	1.12265E136	0.02691	2.15E46
τ_2	0.00104	4.257E-6	332656	198361	0.00852	0.00231	1.232E10	1.7693E11	0.00524	0.00299	117.36388	31.75647	190.93415	108.94456	0.00591	0.0016
R^2	0.99	0.991	0.991	0.993	0.992	0.99	0.94	0.99	0.991	0.993	0.993	0.991	0.989	0.989	0.99	0.97

YPO_4:10%Yb^{3+}, 50%Nd^{3+} RE^{3+}:$(PO_4)^{3-}$ ratio =5																
y_0	9.148E-4	7.035E-6	0.261506	0.341	0.00714	0.70339	1.9664E7	0.112	0.00714	0.60576	140.05497	896490.0585	140.04737	11880.95842	0.00495	31.6791
A_1	9.809E-4	6.454E-6	4.161E11	5.2E11	0.00469	2.0438E-4	4450.2524	3242.84144	0.01142	5.58952E-4	213.00273	9.27312	87.53954	4.28334	0.00325	1.4167E-4
A_2	7.235E-4	1.893E10	0.76961	5.4E13	0.03728	6.018E11	-0.00484	3.7027E65	0.0012	7.75257E62	26.82532	4.33069E14	832.97024	5.37904E68	0.02584	4.17E11
τ_1	6.701E-4	4.380E-6	2.4132E9	6.69E8	0.00577	6.821E-5	31.45145	19.45901	0.01664	9.71323E-4	173.18183	2.04586	60.10735	3.50929	0.004	4.728E-5
τ_2	7.060E-4	4.277E-6	188.618	131.128	0.01419	7.979E-4	5.4068E9	1.70183E9	0.00562	7.4001E-5	70.46028	3.96133	178.07153	2.34653	0.00984	5.530E-4
R^2	0.991	0.99	0.99	0.995	0.991	0.99	0.989	0.987	0.99	0.991	0.994	0.99	0.991	0.992	0.991	0.99
YPO_4:10%Yb^{3+}, 1%Nd^{3+}																
y_0	0.00909	4.2303E-5	0.49543	0.01324	0.03352	5.0101E-4	0.13983	3.87701E-4	0.69202	0.00235	29.83184	0.44587	1.44504	0.0049	0.02324	3.4727E-4
A_1	0.00826	4.0834E-5	0.48647	0.01374	0.03268	5.0576E-4	0.16694	3.78611E-4	0.68069	0.00189	30.59718	0.47349	1.46909	0.00407	0.02265	3.5057E-4
A_2	0.00889	3.4061E-5	0.45731	0.01469	0.03096	5.3893E-4	0.21391	4.70429E-4	0.49103	0.00117	32.30381	0.5624	2.03653	0.00486	0.02146	3.7356E-4
τ_1	0.00406	1.4572E-5	1.55966	0.02492	0.02338	2.5835E-4	0.19459	0.00467	0.0861	9.1154E-4	42.77666	0.47275	11.61404	0.12295	0.0162	1.7908E-4
τ_2	0.00418	1.3301E-5	1.49192	0.0176	0.02516	2.7991E-4	0.21436	0.00691	0.07756	9.61413E-4	39.75095	0.44231	12.89349	0.15983	0.01744	1.9402E-4
R^2	0.99	0.99	0.991	0.991	0.989	0.992	0.995	0.992	0.99	0.989	0.991	0.992	0.99	0.991	0.992	0.993
YPO_4:10%Yb^{3+}, 25%Nd^{3+}																
y_0	8.3959E-4	3.3229E-4	0.06692	0.07212	3.50062E-4	0.0161	0.08247	0.076	0.52595	0.023	2856.64001	0.31	1.90132	0.213	2.4264E-4	2.4264E-4
A_1	0.00279	1.9540E-5	0.09053	0.00178	0.14642	0.00159	1810.1643	44.05908	0.01523	4.38994E-5	6.82971	0.07402	65.65704	0.18924	0.10149	0.10149
A_2	0.00191	1.6753E-5	0.07162	0.00118	0.16096	0.00157	2788.9404	57.76091	0.01452	3.37328E-5	6.21291	0.06062	68.88981	0.16009	0.11157	0.11157
τ_1	0.00116	9.5822E-6	6909.5707	5.731E7	0.0135	0.09667	180.24707	5.73152E7	0.0135	3.7019	74.09665	530.77063	74.08714	20319.38446	0.00935	0.00935
R^2	0.998	0.991	0.99	0.99	0.991	0.989	0.988	0.99	0.992	0.99	0.991	0.991	0.992	0.99	0.992	0.989
YPO_4:10%Yb^{3+}, 75%Nd^{3+}																
y_0	0.00326	3.2027E-5	23.15273	0.52672	0.03037	1.1844E-4	506449.86	61299.00863	0.0087	7.75566E-5	32.9238	0.1284	114.95532	1.02489	0.02105	0.02105
A_1	0.00355	3.2806E-4	18.41542	1.18349	0.03503	4.9311E-4	30.5467	5.30006E11	0.00452	2840740.6441	28.54869	0.40191	221.48242	1.39351E11	0.02428	0.02428
A_2	0.00293	2.34E-5	51.23647	1.28343	0.02542	9.3372E-5	1255973.8	148485.628	0.00816	6.72847E-5	39.3359	0.14448	122.54189	1.01038	0.01762	0.01762

**τ_1**	0.00193	1.9730E-5	2.99676E7	2.11E11	0.00701	0.22915	856200	2.1135E11	0.00701	8.00997	142.67146	4664.30424	142.66675	163033.35489	0.00486	0.00486
**τ_2**	0.00326	3.2027E-5	23.15273	0.52672	0.03037	1.1844E-4	506449	61299.00863	0.0087	7.75566E-5	32.9238	0.1284	114.95532	1.02489	0.02105	0.02105
**R^2**	0.992	0.992	0.991	0.99	0.99	0.987	0.99	0.992	0.993	0.99	0.991	0.989	0.982	0.99	0.991	0.99

Temperature determination uncertainty was calculated as follows:

$$\delta T = \frac{1}{S_R} \frac{\Delta \tau_{avr}}{\tau_{avr}} \qquad \text{(S3)}$$

where $\Delta\tau_{avr}$ represents the change of the τ_{avr} corresponding to ΔT.
